# Precaution, Responsible Innovation and Beyond – In Search of a Sustainable Agricultural Biotechnology Policy

**DOI:** 10.3389/fpls.2018.01884

**Published:** 2018-12-18

**Authors:** Alexander Bogner, Helge Torgersen

**Affiliations:** Institute of Technology Assessment, Austrian Academy of Sciences, Vienna, Austria

**Keywords:** biotechnology policy, European Union, GMO regulation, gene editing, Precautionary Principle, Responsible Research and Innovation

## Abstract

The recent ruling by the European Court of Justice on gene edited plants highlighted regulatory inadequacy as well as a decades-old political problem, namely how to reconcile diverging expectations regarding agricultural biotechnology in Europe. Over time, regulators had tried out various tools to address concerns and overcome implementation obstacles. While initially focussing on risk (with the Precautionary Principle), they later tried to better embed technology in society (e.g., through Responsible Research and Innovation). The PP got criticized early-on; meanwhile, it seems to have lost much of its salience. Responsible Research and Innovation (RRI) is associated with problems of participation and political impact, often rendering it a public awareness tool only. We discuss problems with both approaches and conclude that also RRI falls short of facilitating technology implementation in the way regulators might have had in mind. Rather than leaving political decisions to technical risk assessment or ethics and public awareness, we argue for re-establishing a broad yet sober process of opinion formation and informed decision-making in agricultural policy.

## Introduction

The European Court of Justice’s ruling (Court of Justice of the European Union [ECJ], 2018) that gene edited crops should be assessed like traditional genetically modified organisms (GMOs) elicited split reactions. While some scientists criticized that it jeopardized the future of plant breeding in Europe (Stokstad, 2018), others lamented other scientists’ hypocrisy (Stirling, 2018). NGOs greeted it in the name of consumer rights (Friends of the Earth [FoE], 2018). The comments not only suggest regulatory inadequacy but also show how deeply split stakeholders are over the future of agricultural biotechnology in Europe. They seem to agree, though, that gene editing is a game changer, offering unprecedented opportunities for achieving new traits without introducing foreign DNA. Since distinguishing gene edited from ‘naturally’ bred varieties will be difficult, the technology might be a vehicle for bringing crops with targeted genetic alterations onto the field – a relief for some and a nightmare for others. However, it remains unclear how the ruling can be implemented.

The European Court could have followed the more relaxed proposal by the advocate-general, who argued that gene editing should be exempted because there is no new DNA in the plant (Abbott, 2018). However, the Court focussed on the ‘lack of experience’ with targeted alterations of the genome, in contrast to the results from older methods of mutagenesis that had proven safe. Thus, the Court used the same argument as applied for regulating recombinant DNA long ago. Today however, in the light of 30 years of safe use, the latter might be considered safe as well. Since it is not considered as such, it is unclear which amount of experience will be held sufficient to exempt a technology from additional scrutiny in the future. After all, gene editing is one of the latest innovation to challenge European regulation but probably not the last.

Whilst regulatory inadequacy is a problem in itself it highlights a bigger political problem: how to reconcile diverging demands and expectations regarding agricultural innovation among the European Union’s stakeholders, institutions and member states. It is by no means a new problem as regulators had to learn early that agricultural biotechnology would not proceed in a business-as-usual way. For decades they strived to ‘make biotechnology happen’ (Torgersen et al., 2002), promoting innovation by generously supporting research and development toward economic applicability together with ensuring safety by providing restrictive risk regulation (Jasanoff, 1995). Thus they tried to meet widespread concerns that impeded the implementation of biotechnology.

This double strategy was not without problems: “Obviously, governments thought that biotechnology was something worth developing and they supported it with alacrity. Yet they also styled themselves as impartial regulators of what many perceived to be a risky endeavor. This ambiguity later proved to be one of the sources of public distrust” (Torgersen et al., 2002, p. 23). Despite all research support and risk regulation, the European public could never be convinced of the advantages of agricultural GMOs^1^. Efforts spent on understanding the background for public skepticism (e.g., Gaskell et al., 2004, 2010) made it clear that the underlying reasons are complex and often prone to misinterpretations^2^.

Over time, regulators came up with a variety of innovative policy tools to address the conundrum, in their view, of public concerns and thus to overcome the obstacles to technology implementation. The initial focus on risk mitigation ran into difficulties as it proved to be too narrow. Later, it was supplemented by a broader approach aimed at anchoring technology in society. However, both attempts had their particular problems and eventually failed. We claim that analyzing the role of these tools provides a fruitful analytic perspective to distinguish different attempts at ‘making biotechnology happen’ that may also influence future endeavors in this respect. From such a perspective, we argue for shifting the emphasis from regulating the technology to pursuing comprehensive agricultural policy goals.

Following this rationale, the article will focus on the Precautionary Principle (PP) as a tool to mitigate uncertain risks and, more contemporary, on Responsible Research and Innovation (RRI) as a value oriented concept to anchor technological development in society. Although the PP and RRI have little in common content-wise, we think they shared a political function, albeit using different strategies: they both should prevent or bring down controversies over particular applications among stakeholders and the public. These controversies were seen as the major obstacles to the implementation of biotechnology (i.e., to ‘make biotechnology happen’). In the context of the political and regulatory efforts to overcome controversies, the PP’ rationale appeared as that of an ‘emergency brake’ in (rare) cases of unclear but potentially severe risks. While it was intended to reassure critics it fostered, in practice, a rhetoric of scientific risk arguments and their dismissal. We will address how this narrow focus proved insufficient to address the underlying concerns and how the PP eventually became the target of criticism itself.

When the attention turned to new areas like nanotechnology that seemed prone to elicit similar controversies, a broader approach appeared necessary that transgressed the boundaries of technological risk assessment and addressed societal issues as well. Over time, attempts concretised under the umbrella of ‘Responsible Research and Innovation’ (RRI)^3^. It catered to shortcomings of previous attempts to foster cooperation rather than conflict in various ways: (i) Since the authorisation process proved to be too late a step for leverage, activity sets in much earlier. (ii) The concept of mission orientation appeared handy to align innovation with ‘grand challenges’ addressing major contemporary problems. (iii) Societal preferences as they emerged from public debate are taken into account, together with, and framed by, established ethical principles and normative frameworks. (iv) Rather than in a top-down way, technology development is reconciled with societal values and expectations through participatory procedures. The rhetoric exceeded the narrow focus on risk; however, and despite considerable efforts at defining, fleshing out and implementing RRI through big EU funded projects,^4^ it remained a framework providing orientation at best rather than becoming a policy principle.

Since both tools with their respective reference to risk or ethical principles and societal values could not sustainably cope with the recalcitrant problems of ‘making biotechnology happen,’ the question now is how to proceed in the light of technologies like gene editing. Since business as usual does not seem feasible, we will finally ask how a solution could look like. In our view, the regulatory orientation at the technology must be revised in favor of a goal-oriented comprehensive agricultural policy emerging from an open political process of EU-wide opinion-formation among stakeholders and society at large, difficult as it probably will be.

## Precaution or the Transformation of Political Disputes Into Risk Issues

### The Precautionary Principle and Its Double Role in Risk Controversies

While most new agricultural technologies did not raise much concern, the genetic modification of crops triggered questions of safety and risks, benefits and their equitable distribution long before the technology was put into practice. In the late 1980s, risk claims might not have been surprising: with little experience, it was still unclear whether the new breeds would behave as predictably as traditional ones. Scientifically determined health and environmental risks, if evident, usually entail regulatory action, so technology critics tried to prove such risks, though largely in vain. Technology supporters considered speculations about risks as unscientific and demanded sticking to positive evidence as the only legitimate basis for regulation (Miller and Conko, 2001). Nevertheless, in the absence of conclusive evidence any remaining uncertainty perpetuated risk claims (Bourrier and Baram, 2011). Mitigation tools failed to solve the conflict because a variety of other fears looming behind took the shape of risk arguments (Gaskell et al., 2004).

During the 1990s, the European Commission took up previously existing ideas of precaution and reformulated them. The PP in its then new form became the hallmark of European risk regulation. It addressed a pressing problem: if there are strong hints at a risk but experts disagree about its presence, magnitude or cause, long legal battles and an unacceptable delay in regulatory action might ensue. In some cases such as tobacco and asbestos, this had caused unnecessary uncertainty and a high death toll (Harremoes et al., 2001). Here, it would eventually prevent particular risk-prone applications of the new technology from being implemented. The PP might provide a regulatory shortcut in those (rare) cases where there are strong indications but no full evidence of a severe risk (Von Schomberg, 2013), provided that there are cost-effective ways to reach the desired aim of risk reduction^5^. Thus, the principle of uncontested scientific evidence as a precondition for case-specific regulatory action became questioned. To allow sorting out the few cases where the PP might apply from the vast majority of others the notion of uncertainty got further specified, integrating risk assessment into a ‘precautionary process’ (Stirling, 2007). Despite such attempts at sophistication, the temptation to apply the PP as a last resort in cases where a product was unwanted remained: in a number of trade-related conflicts, the EU, referring to the PP, tried to prevent the import of food products with the argument of health risks (e.g., Millstone et al., 2004). These cases highlighted the propensity to political misuse that critics always had feared.

In contrast, the political intention might well have been that the PP should facilitate technology implementation by reassuring critics that no risks had to be feared as preventive action would be taken even if full evidence was lacking. For example, the European Directive 90/220 on the Deliberate Release of genetically modified organisms (European Council, 1990) made precaution mandatory, emphasizing the safe use of the technology. When the then new Gene Technology Law was debated in 1992, the Austrian Parliament demanded that any application should be made subject to the PP; it therefore went into the preamble (Österreichischer Nationalrat, 1994). It was a concession to the critics to ensure a safe and smooth introduction of the technology. However, the PP was often understood as reversing the burden of proof, which manifested in preventing any deliberate release or marketing of GM products. The political basis for such an understanding was a widespread public aversion against GM crops and food, effectively orchestrated by environmental groups, some farmers and big retail companies (Lassen et al., 2002)^6^.

The lesson learned was that referring to the PP in a political way proved to be effective to halt a technology. Among innovation conscious policy makers (especially in the United States) the PP therefore became anathema^7^. Everybody thought over twice before invoking the PP in a concrete case because this could have unpredictable consequences. Intended as a pragmatic means to evade long and futile legal battles, the PP had been turned first into a policy tool to reassure critics that risk would not be tolerated so that the implementation of a contested technology could proceed. In a second step, it resulted in severe obstacles to technology implementation and innovation – even if not invoked. Regarding GMOs, namely, its impact was symbolic and political rather than contributing to mitigate risks in practice.

The attempted policy function in managing the controversy – precautionary action to calm critics – had a perverse effect as disputes over the appropriate interpretation and application of the PP itself became part of the debate (Van den Daele et al., 1996). Rather than providing a solution to the on-going conflict, the interpretation of the PP opened up a novel turf that mirrored local idiosyncrasies in member countries (Levidow and Carr, 2005), where preferences on how to deal with agricultural biotechnology differed^8^. On the EU level, the incongruent assessment manifested in conflicts over the market approval of GM crops and, consequently, in diverging voting behaviors of the competent ministers in the European Council. Analyses showed that voting mostly depended on political factors such as public opinion or the government party line (Mühlböck and Tosun, 2018). Attempts to solve the issue on an EU level therefore ended up in a limbo as member countries could not agree on a common policy (Hampel et al., 2006). Eventually, a revision of the Directive allowed national governments to ban GM crops temporally (European Parliament and European Council, 2015). Thus, the EU regulation including the PP had (almost pathetically) failed to mitigate risks while ensuring harmonized innovation in a functioning common market.

As a result, and in contrast to other technologies having become less controversial over time, the conflict over GMOs petrified. Official debates over alleged or uncertain risks together with public mobilization and the reluctance of European food retailers to offer GM products efficiently halted the technology. This stalemate has not changed despite an ever more sophisticated regulation.

One reason was that a variety of concerns built on different framings of the issue (Bogner and Torgersen, 2015), to the effect that opponents and proponents lacked a common basis of understanding. The perpetuated administrative focus on risk did not help much as it prevented politics from developing a broader political perspective to reconcile different interests and world views, which might have addressed underlying problems better (Levidow and Carr, 2010). In the meantime, the battle over GMOs in agriculture and food became paradigmatic for controversies mixing risk and non-risk arguments that were expected to arise over other novel technologies. Even if they never manifested, technology developers came to fear them (Rip, 2006; Torgersen and Schmidt, 2013). Since the PP had clearly impeded technology implementation, another way to address concerns in the absence of evidence of risk was deeply needed – not only to solve the GMO conflict but for innovation in general.

### Transgressing the Narrow Focus of Technological Risk

Over recent years, the PP seemed to have lost salience as a risk management tool^9^. Yet the problem of uncertain risks from novel developments remained. For example, experts from three risk evaluation panels of the European Commission identified considerable uncertainty over safety and security from Synthetic Biology (SCENHIR/SCHER/SCCS, 2015). Accordingly, gene drive experiments could pose particular risks to the environment. Radically novel traits or modifications of animals and human beings might bring deep-rooted dreams and fears nearer to realization. ‘Xenobiology’ – unpredictable foreign forms of life incorporating new chemical components – appear possible. Synthetic biology might also render itself to do-it-yourself activities raising serious security and safety issues. One could have expected the PP to play a certain role in their conclusions; rather, they laid emphasis on not foregoing potential benefits from overestimating risks while taking up concerns among the public. Their advice becomes somewhat understandable in the light of the debate on the PP itself.

Not only social scientists had long suspected that risk and its perception is a political issue. Early on, critics of the PP had found the principle to be socially biased as it is said to be sensitive to risks associated with technological change or ecological interventions while being blind for risks from regulation (Sunstein, 2003). Accordingly, this is due to the ‘selectivity of precautions’: the publics (and eventually politics) in different countries are sensitive for particular risks and not for others, subject to national patterns of cultural value preferences^10^. As a result, precaution fosters regulation only if the risk addressed is politically relevant. Therefore, the PP fails to reduce overall risks as it ignores some of them. For example, avoiding potential environmental or health risks by prohibiting a technology does not away with risks from older competing technologies and, in addition, may entail new risks from regulation, if only indirectly^11^.

As an answer to frequent criticism, the European Commission proclaimed an ‘innovation principle’ as a counterweight to the PP, intended to repair its (political) shortcomings (European Commission, 2016). While the PP emphasizes risk, the innovation principle focusses on the opportunities of a new technology, to which any risk should be compared. If a technology would not be implemented due to potential risks, this should be weighed against the benefits forgone, such as avoiding known risks from technologies replaced. Together, it was argued, both principles would adequately represent the double face of technological innovation, balancing the risks when implemented with those when not. If in doubt, the benefits from innovation may weigh heavier as risks are speculative. In practice, however, putting up risks and benefits from old and new technologies against each other is rarely done, so the impression prevails that the innovation principle’s role, too, is mostly symbolic.

Taken together, the focus on risk is subject to political and cultural preferences while seemingly promising objectivity. Reports on the social psychology behind the debate over GM food have shown that the rejection mostly originated from a fear of the ‘unnatural’ and hybrid as a result of the technological tinkering with food (Gaskell et al., 2004; Wagner et al., 2006). In this light, it becomes understandable that the rejection of GM crops and food remained a social fact irrespective of arguments. As a consequence, Sunstein (2003) demanded that technology governance should aim at a better policy to address a broad range of societal concerns as well as benefits including, but exceeding, risk aspects.

## The ‘Participatory Turn’ in Technology Policy – Governing Innovation Responsibly

### The New Mission Orientation in (Bio)technology Policy: From ELSI to RRI

While the EU tried to overcome the GMO conflict by developing and refining a precautionary handling of potential risks, attempts to develop new technologies in line with social values and expectations gained salience and prevail today. Such an orientation at a mission aims at addressing societally and/or economically relevant benefits from technology application and finding ways to realize them (Mazzucato, 2017). It sees a genuinely political task in determining which benefits should be addressed in whose interest. Thus, political action not only pursues the classical task of protecting people from risks. Rather, it aims at the conscious or planned design of innovation and at political impulses for the development of marketable technologies through, i.a., research funding. Rather than being realized top-down, technology will be implemented through governance approaches that build on a network of actors including politics and business, science and civil society.

Dedicated mission orientation emerged after World War II with the era of ‘Big Science,’ leading to success through collaborative work and large resources. The resulting technologies might not have been developed via private initiative or normal scientific progress (Gassler et al., 2006), such as nuclear power, space exploration, semiconductors and, later, ICT or bio- and nanotechnologies. Classical mission orientation contributed to making innovation paramount: “Governments have made of technological innovation an instrument of industrial competitiveness, world leadership, and national wealth.” (Godin, 2016, p. 548).

With a focus on innovation, a new mission orientation was developed that not only focuses on profitable products but also on pressing societal problems (‘Grand Challenges’), with sustainable development as a cross-cutting issue. Value questions such as the responsibility for consequences and non-technical, especially ethical criteria for decision-making are taken into account. Advisory bodies such as the National Ethics Council or the Council for Sustainable Development in Germany illustrate their (symbolic) salience^12^. Rather than eliminating risks, the aim is to implement innovations by reconciling technological development with societal values and expectations.

This approach is condensed in the principle of Responsible Research and Innovation (RRI). It has become a reference point in the debate on governance through a number of EU research projects and policy initiatives^13^. The term had been coined during the 2000s when the controversy over nanotechnology was prevalent. In their ‘European Strategy for Nanotechnology’ the European Commission (2004) defined responsible development as a deliberative process based on the idea that nanotechnology could be guided by ethical principles and solutions, whenever appropriate, should be enforced through regulation. Since then, the European Commission, EU Member States and associated countries have launched various initiatives and activities under the header of RRI. It has been institutionalized as a cross-cutting issue under Horizon 2020, the EU research framework program 2014–2020.

More than 250 articles covered RRI from a social sciences perspective and provided numerous definitions (Burget et al., 2016). Since 2012, René von Schomberg’s influential take appeared in several EU calls on ‘Science with and for Society’:

“Responsible Research and Innovation is a transparent, interactive process by which societal actors and innovators become mutually responsive to each other with a view to the (ethical) acceptability, sustainability and societal desirability of the innovation process and its marketable products (in order to allow a proper embedding of scientific and technological advances in our society)” (Von Schomberg, 2013, p. 63).

What ethically acceptable, sustainable or socially desirable means remains contested, though. In a pluralistic society, normative criteria cannot be defined *a priori* in a technocratic manner, rather, they have to be deliberated by a broad range of societal actors (Stilgoe et al., 2013). As a stopgap, Von Schomberg (2013) referred to normative anchors as stated in Article 3 of the European Treaties. Furthermore, a set of common denominators cut across all the different understandings. According to the extensive review by Burget et al. (2016), three aspects are to the fore:

– A focus on values: Most definitions and frameworks affirmatively refer to moral and ethics in technology issues. Even though more precise definitions are lacking, the call for ethics implies that technology development and innovation should be aligned with the values, needs and expectations of society in order to be acceptable, sustainable and societally desirable.
– Ethics as a design element: In previous technology controversies, the call for ethics was associated with the idea of taming or restricting innovation. Ethics was practiced reactively; it was intended to prohibit unwanted consequences after the innovation had been developed or products had entered the market. With RRI, ethics is referred to as a design element shaping innovation responsibly and proactively rather than an ex post evaluation tool as before. Hence, RRI deliberately uses ethics and ethical arguments to shape technology rather than clinging to a particular ethical tradition itself.
– Public participation: With a more prominent role of value question, the innovation agenda is opened up for all kinds of expertise and experience. In technology controversies focusing on risk, primarily expert knowledge is deemed legitimate. While the focus on risk privileges expert authority, taking other aspects (justice, exclusion, inequality, marginalization, privacy) into account gives stakeholder and lay knowledge a greater role. Therefore, the focus on value questions goes along with an invitation to a variety of actors to participate in the innovation process.

Participatory governance had its precursor in debates around the Human Genome Program on ethical, legal and societal implications (ELSI) that were expected to materialize as soon as the genome sequence would have been established. It served as a blueprint for similar programs on other emerging technologies. From 1994 on, the European Union provided research funding for ethical, legal and social aspects (ELSA) of emerging technologies only to abandon the term two decades later in favor of RRI (Zwart et al., 2014). The main difference laid in the emphasis on socioeconomic impacts such as valorisation, employment and competitiveness. Nevertheless, RRI became charged over time with aspirations at a more democratic and social responsive technology development (Stilgoe et al., 2013).

All this was not intended as a replacement for the PP, although RRI also stipulated that potential risks should be identified early and dealt with in a ‘responsible’ way. Rather than hindering a potentially risky product from being marketed, the process should prevent such a product from being developed at all or ensure that potential risks were catered to during development. The political function was to pre-emptively address potentially disruptive issues in a public debate over newly emerging technologies, be they concerns over risk, ethical implications or societal misfit. Aligning innovation with societal goals and making it ‘responsible,’ so the hope, would take the steam out of a pending controversy and foster technology implementation.

### The Grand Challenge of Public Participation

The focus of RRI on values and ethics immediately suggests a focus on public engagement, because value conflicts and ethical questions cannot be decided on the basis of expert knowledge alone. Vice versa, the emphasis on public engagement for designing innovation indicates that distributed intelligence, pluralism and dissent may have a constitutive role. With research, the tendency toward inclusion is reflected in the concepts of Citizen Science (Irwin, 1995), transdisciplinarity or Mode 2 science (Gibbons et al., 1994). With regard to technology, it manifests in various forms of technology assessment (TA) such as participatory, constructive (Schot and Rip, 1997) or ‘real-time’ TA (Guston and Sarewitz, 2002).

In the context of RRI, however, the status of public participation goes beyond that in TA. In participatory or constructive TA, citizens, consumers and stakeholders participate in isolated events conceived as participation projects (Bogner, 2012). They are non-binding and provide complementary information about citizen values rather than being part of the innovation process itself. RRI, in contrast, promotes formats that enable the continuous involvement of relevant actors. The objective is to institutionalize and routinise public participation in research and innovation (Owen et al., 2013). Attention is paid to heterogeneity, taking into account a large number of divergent perspectives and actors such as stakeholders (NGOs, industry, trade unions, science communication), policy and administration (parliamentary commissions, research funding) and academia (universities, non-university research). In addition, the broader public (as constituted topic-specifically for a participatory event) must be involved. Added value, so the hope, comes from a multiplication of perspectives, a consideration of alternative rationalities and knowledge forms as well as an opening of decision-making processes.

However, there are severe challenges to participation, especially with respect to emerging technologies, along several dimensions: with regard to (1) social aspects, (2) the ‘issue framing,’ i.e., how to discuss what, (3) the timing of an event and (4) the definition of the problem to be addressed.

(1) Regarding social aspects, public engagement requires a panel with a balanced composition of participants, taking into account gender as well as representing various societal perspectives (Rask et al., 2016). At least, particular interests or perspectives must not dominate the deliberation process. To ensure balance, the actors invited should represent a diversity of values and forms of knowledge. RRI also requires a comprehensive, objective (‘balanced’) view upon the issues. The assumption is that participants (especially stakeholders) enter the process holding preconceived interests and views and reproduce the usual conflicts. As a remedy, Von Schomberg (2013) demanded that stakeholders should transcend their stereotypical arguments and strategies – industry representatives should not only highlight economic benefits, NGOs not only risks; rather, they should see the world through the eyes of the other, respectively. However, it is unclear why a stakeholder should forego a short-term benefit from pursuing his or her self-interest in exchange for fostering public welfare-oriented responsible innovation. Institutionalizing public participation in the spirit of RRI therefore demands changing established power relations and conflict structures. Another practical problem is that participants often experience social difficulties in participatory procedures. For example, they are not accustomed to tolerate opinion pluralism or the obligation to provide reasoned arguments. If discussions turn controversial (e.g., on values or identities), those with extreme positions often feel inadequately represented and may leave the group (Bogner, 2012).

(2) With regard to the issue-framing, participation entails prioritizing value questions over questions of knowledge or interests. RRI therefore tends to change the focus from risk to ethics. Risk discourses privilege expert knowledge because claims for health or environmental hazards must be backed with scientific arguments. Ethics that is not limited to medical issues (see Beauchamp and Childress, 1994) includes aspects like justice, exclusion, privacy, marginalization, etc., which extends the range of issues beyond those of risk. In practice, however, standard arguments are rarely exceeded because members of a ‘participation industry’ (experts and institutions from science communication, STS and TA) usually initiate and organize processes from outside. In addition, and especially with new and emerging technologies, participants are rarely involved in the issues at stake and lack the necessary knowledge. Therefore, organizers must define the problems in advance, running the risk of marginalizing alternative problem-solving perspectives (Sykes and Macnaghten, 2013, p. 100). Emerging technologies have not yet found many concrete applications that elicit concerns or hopes. To make due, organizers use analogies, i.e., established problem-solving perspectives from comparable controversies, again marginalizing alternative perspectives. For example with synthetic biology, concrete hopes reared by science and industry prevailed in British participation processes (Bhattachary et al., 2010). In contrast, questions were rather generic: how can synthetic biology contribute to the bio-economy? Can synthetic biology solve the antibiotic crisis? etc. Finally, whether stakeholders are willing to participate depends on the scope of the event. Any seeming indication of lopsided criticism or euphoria jeopardizes a balanced representation (Stilgoe et al., 2013).

(3) Regarding timing, RRI demands early and continuous participation. From 2000 on, nanotechnology triggered a plea for ‘upstream involvement’ (Kurath and Gisler, 2009). Arguments for early participation were derived from the idea that the path from basic research to application was not linear, since technical feasibility and marketability would influence basic research already. Decisions over the path the technology would take were made early, therefore, so the argument, participation must also set in early to render it effective. Thus, the concept of technoscience (Latour, 1987) also fostered early participation. However, few applications exist at an early stage that cause conflicts or inspire the public’s imagination; consequently, few people show interest and people have to be motivated. They are more interested if the subject is relevant to their everyday life or if it is controversially discussed in the media. Then, however, the trajectory usually cannot be changed anymore^14^.

(4) Regarding problem definition, the lack of popular perspectives may either lead to the debate remaining very concrete, with the researchers’ motivations and problems of laboratory processes as subjects. Alternatively, the discussion turns to the meta-level, where general considerations on technology and democracy or the future of participation dominate^15^. Thus, the deliberation runs the risk of remaining abstract, little committed or expert dominated, with normative dissent often remaining implicit (Felt and Fochler, 2010) – participants debate on the meta-level how to responsibly discuss ethics. With positions remaining largely consensual, their main concern is whether all relevant aspects are getting represented. Rather than being advocates of the common good or of perspectives based on their personal value bases they see themselves as service providers: the task is to contribute to the success of a project.

Regarding gene editing, the current EU framework program supports several projects investigating potential implications under the perspective of RRI. However, it is not always clear what exactly to discuss. Subject to the concrete application this must be decided from case to case. Whether the focus on the technology makes sense is therefore questionable. Another problem is the difference between a discussion under RRI and under the PP. As risks should be considered under RRI as well, a usual subject of the debate is whether there are any risks and what they would entail. In the light of the skepticism in some countries, the results of a participation event may therefore not fundamentally differ from that of many previous exercises on GMOs. The hope that technology implementation would be facilitated thus appears futile.

## Conclusion: Where to Go for a Sustainable Policy?

Both the PP and RRI had a role in the (non)implementation of agricultural biotechnology in Europe, although they proceeded from opposite angles, addressing different aspects of the development and operating at various stages in the implementation process. Yet, regarding their common function of ‘making technology happen,’ both show a disappointing performance.

The PP turned out to prevent not only risky developments but the implementation of the technology in general. Designed as a last resort tool to ensure that ambiguous risks would not lead to endless court trials and block the technology as such, it was applied when political decisions appeared impossible to defend. Referring to the PP allowed actors to use seemingly scientific arguments that nevertheless were politically grounded. The PP may have been intended as a reflexive way of dealing with potential risks; however, the controversy over GM food has never been a risk debate only. Rather, it had many roots like the widespread public unease over current agricultural food production systems. When, in addition, national food idiosyncrasies became jeopardized, organized protest ended up in petrified skepticism^16^. This suggests that risk regulation may be an essential part of the regulatory process but an inappropriate tool to cope with political stakes.

Responsible Research and Innovation was intended to guide research and innovation practice toward societal acceptability while fostering innovation. However in practice, it often ends up in a mere tick-boxing activity filling in research proposals forms or in somewhat futile participatory activities as ends in themselves. Activities to involve stakeholder and the public without real impact on the decisions taken have an unclear remit and mostly serve to introduce bits of new technology to a public that has little to say about it. Referring to ethics and a poorly defined ‘responsibility’ of stakeholders (or even laypeople) does not solve the political problem of organizing the relevant sector, agriculture, in a way that would find support with stakeholders and critical citizens alike.

Both principles seek to tackle a major problem for innovation policy, namely to remove obstacles to technology implementation caused by public skepticism. Both turn out to do so with insufficient means – in an attempt to either dress up politics with scientific arguments or to address political problems with public relation tools fostering awareness among a little interested public. Although both risk regulation or laboratory-like deliberation events having turned out to be the wrong turf, neither the PP nor RRI should be dismissed. Rather, they should be applied to those cases they were intended for, namely the reasonable treatment of uncertain risks and the better alignment of innovation policy with societal values. In addition, the remit of participation must be better defined; current procedures may not yet be adequate to uncover demands and concerns nor cultural value preferences; for the moment, participatory events too often focus on disseminating awareness regarding a new technology.

In our opinion, the most important factor for addressing the underlying problems adequately is comprehensive sectoral policy reaching out to other sectors. After all, issues pertain to agriculture and food production, to research and innovation, trade and various other sectors. Rather than principles in need of interpretation being politically instrumentalized as ‘magic bullets,’ deliberate scientifically supported and democratically legitimized policy may be more adequate to tackle those problems. A major shortcoming from previous policies, however, is their strictly sectoral scope. Since agriculture reaches out to so many areas, an inter-sectoral and multi-level approach is needed. For example, sustainability in agriculture is not only an issue for biotechnology regulation but also for R&D funding, industry development, trade rules, regional policy and many more fields.

Regarding regulatory principles, biotechnology policy has traditionally focussed on regulating the technology. This is one reason why in case of a problem, it easily succumbed to seeming solutions replacing the political with either science or ethics. As a remedy, the prerogative of serious long-term oriented policy needs to be reinstalled and the focus on technology replaced by a focus on the common understanding of the aims agriculture should pursue. This implies that the different tasks of agriculture should be openly discussed and the properties of crop plants adapted accordingly. In other words, properties should not only reflect agronomic and economic parameters but a variety of demands according to the context, which may differ from one place to the other. Whether the respective seed has been developed by traditional breeding, chemical mutagenesis, recombinant DNA technology, gene editing or any technology to come, however, is hardly relevant in this context (see Davison and Ammann, 2017). This does not preclude applying the PP in appropriate cases or addressing a relevant measure under the umbrella of RRI. However, the PP is a risk management tool, while RRI, as the name suggests, focusses on shaping (technological) innovation – perspectives too narrow to tackle all the questions that need to be addressed.

The future of agricultural biotechnology is not an issue for plant breeders and researchers only. It requires a broad debate among many disciplines and stakeholders. A good example was a recent Summer School organized by the faculty of Theology University of Munich, where pertaining aspects could be discussed. New ideas diffuse into mainstream thinking as well, which seems to focusses less on technology and more on real problems now. The European Commission seems aware that the system is not sustainable (European Commission, 2015). More recently, a report on the ‘authorisation processes of plant protection products from a scientific point of view’ by the Commission’s Scientific Advice Mechanism (Group of Chief Scientific Advisors [EC-SAM], 2018) advocated not only scientific reasoning but also extending the scope of arguments to criteria usually held to be ‘political.’ Similarly, a report from UN Environment argued for an extension of parameters to take into account when it comes to measuring the performance of agriculture, including the contribution to sustainability goals (TEEB, 2018). These initiatives highlight the need to discuss, define and agree on the many tasks of agriculture. With clear ends we may devise adequate regulatory mechanisms for the means available. The technology used is only one factor, and a less important one, provided it is safe and effective.

As agriculture is one of the hardest bones of contention among the EU policy fields, such a demand might sound unrealistic. Yet in our view, there is no way beyond an open and transparent process of opinion formation that not only involves stakeholders and the European Institutions but includes scientists, politicians from member countries and, preferably, large parts of the European society. Only if we have a clear vision of future agriculture and its various tasks we will be able to decide over GM crops or the products of any other technology to come.

## Author Contributions

AB and HT contributed equally to the research and the writing of the article and agree to be accountable for the content of the work.

## Conflict of Interest Statement

The authors declare that the research was conducted in the absence of any commercial or financial relationships that could be construed as a potential conflict of interest.

## Footnotes

1 Skepticism seems even to have spread to the United States, see International Food Information Council foundation [IFIC], 2018.

2 For example, the hypothesis of a general ‘resistance to new technology’ out of a lack of knowledge among lay people was dismissed in the 1990s already (Bauer, 1997) but remained popular among scientists and regulators (Rip, 2006).

3 The official website (https://ec.europa.eu/programmes/horizon2020/en/h2020-section/responsible-research-innovation) states: “Responsible Research and Innovation (RRI) implies that societal actors (researchers, citizens, policy makers, business, third sector organizations, etc.) work together during the whole research and innovation process in order to better align both the process and its outcomes with the values, needs and expectations of society.”

4 for example: https://www.rri-tools.eu/de

5 See the formulation in the respective EU Directive (European Commission, 2000), not to be understood as shifting of the burden of evidence. Proving the absence of risk would be intellectual nonsense.

6 In retrospect, political action to prevent agricultural biotechnology in some countries might be considered as an early form of contemporary populism.

7 The United States Administration formulated their own ‘precautionary approach’ based on existing legal instruments, arriving at less restrictive but similar precautionary measures without much resistance.

8 A strong driver of the conflict in the 1990s were various concomitant food scandals such as over BSE. Unrelated from a technical point of view, BSE influenced the GMO case as it “turned ‘mad cow’ into a potent metaphor mobilizing public distrust in regulatory arrangements by linking several policy issues.” (Levidow and Carr, 2010, p. 20).

9 A recent call for proposals under H 2020 addressed an obvious lack of empirical data regarding the salience of the PP vi-á-vis the Innovation Principle. It asks to take stock of the implementation of PP since 2000 in various contexts, analyze the effects of the PP and propose several scenarios for the future of the PP and IP (see https://ec.europa.eu/research/participants/portal/desktop/en/opportunities/h2020/topics/swafs-18-2018.html#fn1).

10 Accordingly, the United States took a highly precautionary approach to risks associated with terrorism, tobacco smoking and universal health care, but not to global warming, poverty and, until recently, obesity. Germany, in contrast, was especially concerned with global warming, nuclear energy, gun possession and the genetic modification of food (Sunstein, 2003).

11 For example, the effort for complying with the regulation of transgenic corps might render the development of regionally adapted varieties unrewarding, promoting seed uniformity and the risk for pest resistance with a global impact.

12 Even if in the reality of research funding the focus on value questions remains symbolic rather than having a real impact, actors have to deal with them, which makes them explicit and opens up new lines of argumentation.

13 The European Commission explained RRI as follows: “Responsible Research and Innovation means that societal actors work together during the whole research and innovation process in order to better align both the process and its outcomes, with the values, needs and expectations of European society. RRI is an ambitious challenge for the creation of a Research and Innovation policy driven by the needs of society and engaging all societal actors via inclusive participatory approaches” (European Commission, 2012, p. 2).

14 The situation reminds of the Collingridge dilemma (Collingridge, 1980): when a new technology emerges, its trajectory can still be influenced but knowledge is insufficient to steer it. When knowledge would be sufficient, the trajectory is set.

15 Meta-level ethics throws up questions like: should we refer to ethics when talking about new technologies? Is it useful to discuss ethical aspects or does it fuel the controversy?

16 However, there are indications that public perceptions slowly change. For example, younger people appear to be less critical (Gaskell et al., 2010) – not only in Europe but also in China (Cui and Shoemaker, 2018).
